# Prophylactic phage administration reduces *Salmonella* Enteritidis infection in newly hatched chicks

**DOI:** 10.1002/mbo3.70002

**Published:** 2024-12-16

**Authors:** Lorna Agapé, Pierrette Menanteau, Florent Kempf, Catherine Schouler, Olivier Boulesteix, Mickaël Riou, Thierry Chaumeil, Philippe Velge

**Affiliations:** ^1^ INRAE, Université de Tours, UMR ISP Nouzilly France; ^2^ INRAE, UE‐1277‐PFIE (Plateforme d'Infectiologie Expérimentale) Nouzilly France

**Keywords:** bacteriophages, chicken, microbiota, prophylaxis, *Salmonella*

## Abstract

Salmonellosis outbreaks are global issues primarily associated with the consumption of poultry products, which may be infected with *Salmonella*. The use of lytic bacteriophages could be a safe and effective approach to reduce *Salmonella* prevalence in poultry and subsequently the incidence in humans. This study examined the value of prophylactic phage treatment on *Salmonella* levels in chickens and the effect of such treatment on their overall gut microbiome. We also investigated phage persistence in vivo and resistance emergence against the six‐phage cocktail used. The preventive potential of phages was evaluated on 200 chicks by administering phages via drinking water for 6 days after hatching, followed by the *Salmonella* Enteritidis challenge on Day 7. The results showed that up to 4 days postinfection, phages had a preventive effect by significantly reducing *Salmonella* colonization in ceca by three logs. Furthermore, the phage cocktail did not induce dysbiosis, although variations in microbiota in terms of microbial composition were observed between conditions, with the Enterobacteriaceae family being impacted. However, the phage cocktail did not induce a long‐term effect, with *Salmonella* levels rebounding 8 days after phage treatment was stopped. Overall, our data show that phage prophylaxis can reduce *Salmonella* colonization and explore ways of improving the effectiveness of phages in limiting infections throughout poultry production.

## Graphical abstract

This study evaluated the effectiveness of prophylactic phage treatment in reducing *Salmonella* Enteritidis levels in chickens and its impact on their overall gut microbiome. Additionally, we examined the persistence of phages in vivo, both in the presence and absence of *Salmonella*, and investigated the emergence of bacterial resistance to the 6‐phage cocktail used.

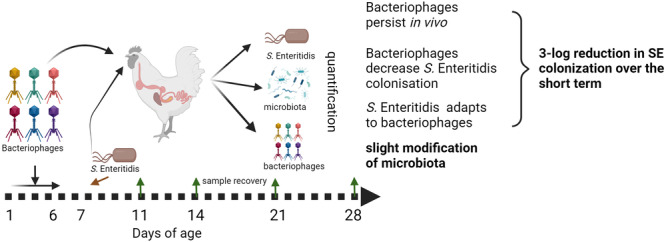

## 1 INTRODUCTION

*Salmonella* is one of the main causes of foodborne diseases and is a serious economic and public health concern throughout the world (Majowicz et al., 2010). Nowadays, non‐typhoidal serotypes, most commonly Typhimurium and Enteritidis, are responsible for more than 70% of human infections in the EU, inducing gastroenteritis and acute diarrhea (EFSA, 2022). The total number of cases of human gastroenteritis due to *Salmonella* is estimated to be 93.8 million per year worldwide and is one of the leading causes of foodborne hospitalization (Majowicz et al., 2010). In 2021, EFSA identified *Salmonella* as the cause of 20.8% of outbreak‐associated cases and 45% of outbreak‐associated hospitalizations in Europe (EFSA, 2022). These foodborne outbreaks are mainly related to poultry product consumption, in particular, through the ingestion of contaminated eggs or poultry meat (Antunes et al., 2016; EFSA, 2022; Shah et al., 2017).

*Salmonella* Enteritidis (*S*E) represents the predominant serovar, associated with 79.7% of all *Salmonella* outbreaks (EFSA, 2022). Indeed, although different serotypes are related to poultry, such as *Salmonella* Typhimurium, *Salmonella* Enteritidis is the most frequent serovar paired with broilers and meat contamination. Nevertheless, other serovars, such as *Salmonella* Infantis have been increasingly associated with broilers and broiler meat (up to 36.5% and 56.7% respectively in 2018) (EFSA, 2019). Thus, protecting poultry from *Salmonella* infection has become an important challenge. However, detection and eradication of *Salmonella* in chickens may be challenging because non‐typhoidal *Salmonella* most often induces an asymptomatic infection that may be accompanied by heavy fecal shedding (Kempf et al., 2022). The pathogen spreads rapidly through the flock once a broiler chick is infected, and the main control measure at this step remains mass culling, or full elimination, of the flock, which is associated with significant economic losses to the poultry producers in the EU (Europe, 2003).

In addition to biosecurity practices, vaccines are one of the main measures used to prevent *Salmonella* infections in poultry (Desin et al., 2013; El‐Saadony et al., 2022). However, they may not be very effective in young chicks in terms of degree of protection, varying from chick to chick due to the immaturity of their immune system (Desin et al., 2013). Antibiotics were previously commonly used to prevent *Salmonella* infection in poultry and subsequently human contaminations (Castanon, 2007; Cogliani et al., 2011). However, their excessive and preventive use has led to the emergence of multidrug‐resistant bacteria and subsequently to their current prohibition in Europe (Castro‐Vargas et al., 2020; Cogliani et al., 2011). It is therefore necessary to develop new strategies to control *Salmonella* infection in chickens. In this context, phage therapy appears to be a promising strategy to prevent and treat infections in the poultry industry (Moye et al., 2018).

Bacteriophages (phages) are viruses that specifically infect bacterial cells. Their specificity presents a real advantage over antibiotics, which have a much broader spectrum of action, due to their specificity as it allows phages to target specific bacterial species without strongly altering the microbiota. In recent years, various studies were conducted to reduce the *Salmonella* load in poultry by the use of bacteriophages (Khan & Rahman, 2022; Wernicki et al., 2017). A previous study reported that phages active against *Salmonella* serovars Enteritidis and Typhimurium reduced cecal colonization of 36‐day‐old birds by 4.2 log10 CFU and 2.19 log10 CFU, respectively, within 24 h, compared with controls (Atterbury et al., 2007). Some studies reported moderate phage effect such as the study of Fiorentin et al. who reported a 3.5‐fold reduction of *Salmonella* Enteritidis in cecal content, 5 days after phage administration but no reduction 25 days after treatment (Fiorentin et al., 2005). Another study reported a reduction in *Salmonella* Enteritidis recovered from cecal tonsils at 24 h compared with untreated controls, and no difference at 48 h posttreatment (Andreatti Filho et al., 2007). Most of these studies on phage therapy have focused on the phage effect in reducing ongoing infection while ignoring other challenges associated with the use of phages (Khan & Rahman, 2022; Ly‐Chatain, 2014). Issues that remain to be investigated include phage delivery to live animals (route, time, and/or frequency of administration), phage abilities to propagate inside the host, bacterial resistance to phage, or, in terms of phage safety, the impact of phages on the gut microbiota (Caflisch et al., 2019; Ly‐Chatain, 2014). It is therefore essential to study these parameters to optimize the effectiveness of in vivo phage treatments. For instance, phage prophylaxis through drinking water merits further investigation as a convenient and easily applied large‐scale treatment to prevent *Salmonella* colonization and its spread over the flock during the critical first stage of production.

This study aimed to assess the ability of a phage cocktail to prevent *Salmonella* Enteritidis colonization of the gut of commercial broiler chickens after oral consumption of phages. We evaluated the general prophylactic use of a phage cocktail containing six lytic phages effective against a range of *Salmonella* serotypes. In this study, as a prerequisite, we first assessed the ability of phages to survive in the intestinal tract of chicks and then tested the efficacy of the phage cocktail in *S*. Enteritidis‐infected chickens. We also determined the effect of this cocktail on the microbiota composition of the chicks during the experimental trial to check for the potential positive or negative impact of phages on the gut microbial composition. Thus, the present study provides further insight into the preventive use of phage by investigating parameters not often tested in this type of trial essential to optimize the effectiveness of phage treatments.

## 2 EXPERIMENTAL PROCEDURES

### 2.1 Bacterial strains and phages used in this study

Two *Salmonella enterica subsp. enterica* strains were used in this study; *Salmonella* Enteritidis LA5 (Menanteau et al., 2018) and *Salmonella* Newport SN388. The *Salmonella* Enteritidis LA5 strain was isolated from broiler chicken and is a nalidixic acid‐resistant and a spontaneous streptomycin‐resistant strain which is characterized in the studies of Grepinet et al. (2012) and Dibb‐Fuller et al. (1999). The *Salmonella* Newport SN388 strain was used for phage titration as it is the strain sensitive to most of the phages of the cocktail. For liquid cultures, both bacteria (preserved in 50% [v/v] glycerol) were routinely cultivated in lysogeny broth (LB, Miller formula) and grown overnight at 37°C at 180 rpm. Six lytic bacteriophages isolated from environmental water and specific to *Salmonella* were used in this study (Table 1). The phage cocktail was supplied by a commercial entity specializing in developing and commercializing phage preparations for phage therapy and other applications. In the cocktail preparation, phages were assigned as SalE_1, SalE_2, SalE_3, SalE_4, SalE_5, and SalE_6 and are present at equivalent ratios. The six phages have a different spectrum of action on *Salmonella* strains (Appendix Table A1), belong to different genera or have different genome sizes (Table 1). Four of them (SalE_2, SalE_3, SalE_4, and SalE_5) have lytic activity on the *Salmonella* Enteritidis LA5 strain and SalE_6 has enzymatic activity being able to lyse bacteria but not to multiply and form plaques in the spot assay.

**Table 1:** Taxonomic classification phages composing the cocktail.

Phages	Source	Family	Sub	Genus	genome size (bp)
SalE_1	Mix of environmental waters, Maryland	Ackermannviridae	Aglimvirinae	*Agtrevirus*	159620
SalE_2	Mix of environmental waters, Maryland	Ackermannviridae	Cvivirinae	*Kuttervirus*	157235
SalE_3	Brackish Water, Maryland	Unclassified	Vequintavirinae	*Seunavirus*	147745
SalE_4	Mix of environmental waters, Maryland	Straboviridae	Tevenvirinae	*Gelderlandvirus*	*164224*
SalE_5	Brackish Water, Maryland	Unclassified	Ounavirinae	*Felixounavirus*	102179
SalE_6	Brackish Water, Maryland	Unclassified	Ounavirinae	*Felixounavirus*	85390

### 2.2 Bacterial and phage titration

*Salmonella* titration from chicks' samples was performed on crushed organ suspensions decimally diluted in Dulbecco phosphate‐buffered saline with Ca^2+^ and Mg^2+^ (DPBS) (Thermo Fisher Scientific) and then spread by spiral plating (easySpiral Pro, Interscience) onto selective *Salmonella‐Shigella* (*SS*) agar medium (Bio‐Rad) on which *Salmonella* is differentiated by the production of hydrogen sulfide (H2S) resulting in black colonies and containing 500 µg/mL of streptomycin to further discard other bacteria (Barrow et al., 2004; Lee et al., 2015). The method used, described in Menanteau et al. (2018), is very sensitive and robust and has been extensively described in (Velge et al., 2022). *Salmonella* detection results were confirmed by slide agglutination tests with monovalent antisera to specific O9 antigens. When no *Salmonella* colonies could be found and quantified, an enrichment procedure was used to detect their presence or confirm their absence in the samples. For this purpose, the remaining suspensions of crushed organs were inoculated in trypticase soy broth (TSB) (BioMérieux, France) and incubated at 37°C overnight. Then, 1 mL of the enrichment culture was spread by spiral plating onto *SS* agar plates and incubated at 37°C for 24 h to detect any remaining bacteria. The detection threshold after enrichment is one bacterium per organ. For positive *Salmonella* detection after enrichment, the *Salmonella* level was fixed at 1 log10 CFU/g on graphs.

To determine phage titers, suspensions of crushed organs were first subjected to centrifugation at 15,000*g* for 10 min to remove debris. The supernatant was then filtered through a 0.22 µm pore size filter to remove bacteria. The filtrates were serially diluted 10‐fold in DPBS and spot assays were performed using a double agar overlay technique with lawns of *Salmonella* Enteritidis LA5 and *Salmonella* Newport SN388. The molten soft LB agarose (0.5% (w/v) containing 10 mM MgSO_4_, 1 mM CaCl_2_, and 30 µM 2,3,5‐triphényltétrazolium chloride) was poured onto 15% (w/v) LB agar plates as previously described (Kutter, 2009). The limit of detection for this assay is around 2 log10 PFU/g depending on the organ.

Plates were incubated overnight at 37°C before enumeration of *Salmonella* colonies that were expressed as CFU/g of organs. Phage titers were expressed as PFU/g of organs.

### 2.3 Phage one‐step growth curves

The one‐step growth curves were performed as described by Kutter et al. (2009), with minor adaptations. Subcultures of *Salmonella* Enteritidis LA5 at the exponential phase were adjusted to yield a cell density of 10^7^ CFU/mL and phages were added to reach a multiplicity of infection (MOI) of 0.1. After 10 min at 37°C of phage adsorption, a 100‐fold dilution was performed to terminate phage adsorption and neglect the percentage of free phages in the culture medium. Following this synchronization step, aliquots were collected every 5 min for 1 h for phage quantification as PFU/mL using the spot assay method. Triplicate experiments were performed for each phage test. Burst size was estimated by calculating the ratio of liberated phages to PFU enumerated before the onset of lysis (Hyman & Abedon, 2009).

### 2.4 Identification of bacteriophages by multiplex PCR

The neat dilutions of clear spots were extracted from the agar overlay of phage counting plates with a sterile toothpick, and the resulting agar plugs from the different samples were pooled and suspended in 300 ml of H_2_O. The tubes were incubated for 1 h at room temperature to allow diffusion of phages into water and then used as a DNA template for the PCR assay.

One pair of primers per phage species was designed from a specific region for each phage (Table 2) using Geneious 10.2.6 software from sequence data of all phages and product sizes obtained ranging from 109 to 941 bp. Primers were ordered from Eurogentec. Every PCR reaction was performed in 50 µL using the Fidelio Hot Start PCR kit (Ozyme) and comprised the six different primer pairs of appropriate concentrations (3 µM for SalE_1, SalE_2 and SalE_3 and 1.5 µM for SalE_4, SalE_5 and SalE_6 forward and reverse primers), 0.2 mM deoxynucleoside triphosphates, 2U of Fidelio® Hot Start DNA Polymerase enzyme, 1x Fidelio® HF Buffer and 5 μl of the DNA templates. The multiplex PCR was performed for 30 cycles as follows: denaturation at 98°C for 10 s, annealing at 65°C for 30 s, and extension at 72°C for 30 s. An additional initial denaturation step at 98°C for 4 min and a final step of extension at 72°C for 5 min were performed.

**Table 2:** Primer sequences for detection of phages present in the cocktail.

Phages	Primers	Primer sequences (5'–3')	PCR product size (bp)
SalE_1	Forward	CGGTACAGAGCATATTGAAGCCCTG	941
	Reverse	CGATGTGTTCGTCCAACAGGAAGTG	
SalE_2	Forward	CTGGAAGAAATGACCTGGGGCAGAT	718
	Reverse	TCTGGGCGCATTAGCTGTATCTCGT	
SalE_3	Forward	CACCAGGTAGACGATCTTCATCGGT	542
	Reverse	TGTTCCAGGATGGTGAAGCGAAGGT	
SalE_4	Forward	TTAACTCCTCCTGCCGGAGAACCAT	340
	Reverse	CTGCTCGGAGATGTTCCTTGGGTTA	
SalE_5	Forward	CGTCCTCAAGTTCTTCCTCTGGGAT	232
	Reverse	GACAAGCCGGAAGGTATCTGGAACT	
SalE_6	Forward	GGCTTTTTTGACCTCGACAAAGGCA	109
	Reverse	GCATGTTGGCGTATGTTGCAAGCCT	

The PCR products were separated on a 2% agarose gel in TAE buffer (Thermo Scientific™), and stained with Midori Green (NIPPON Genetics EUROPE). A single plaque picking was sufficient to generate a positive result by the PCR.

### 2.5 Experimental design

Two trials were successively performed using commercial Ross 308 broiler chickens. Fertilized eggs were obtained from the incubation establishment BOYE Accouvage with hatchings proceeding in confined conditions at the experimental animal infection unit (PFIE, 10.15454/1.5535888072272498e12).

In the first trial, to determine the persistence of phages in the intestine without taking into account recontaminations, after hatching, chicks were raised in isolators, which are experimental breeding systems avoiding cross‐contaminations by constantly changing the air and decontaminating the droppings (Menanteau et al., 2018). In the second trial, chicks were raised in confined rooms and housed in pens on straw litters. All birds received an identical diet. Feed and water were supplied ad libitum with an applied 12:12 L:D lighting scheme. For both trials, phages were administrated ad libitum through tap drinking water diluted to a final concentration of 10^9^ PFU/mL. In this condition, knowing that chicks aged between 1 and 10 days (Brake et al., 1992) drink about 50 mL per day, it is possible to speculate that they should have consumed about 5×10^10^ PFU per day. Chicks' water consumption was monitored during the 6 days of administration by weighing the amount of water consumed per day. Before each experiment, chick feces were recovered to confirm the absence of any pre‐existing *Salmonella* spp. using the selective enrichment Rappaport Vassiliadis media and then Rambach agar (Conda‐Pronadisa, Spain).

#### 2.5.1 Trial 1

The in vivo stability of the phages and their ability to persist in the chicken's gut without the presence of *Salmonella* host was evaluated in the first trial. Fifty newly hatched chicks were raised in isolators over a span of 14 days. Phages were continuously administrated to chicks via drinking water at a concentration of 10^9^ PFU/mL during their six first days of life (Days 1–6). A control group was also included in which the chicks received only water during the trial. Then at Days 7, 15, and 20 (1, 9, and 14 days after phage administration was ended), the crop, gizzard, ileum, cecum, liver, and spleen of 5 chicks per sampling day were collected, weighed, then crushed and homogenized in DPBS using the MiniMix 100 P CC and BagPage filters (Interscience, France). Phages were then enumerated in both groups by spot assay and identified by PCR from these chick organ suspensions as described above.

#### 2.5.2 Trial 2

The preventive effects of phage administration on *Salmonella* infection in chicks were evaluated in the second trial. Two hundred newly hatched chicks were divided into four groups of 50 chicks housed in pens in 4 different rooms for 28 days. The control group (Control) was untreated by phages and unchallenged by *Salmonella*. The second group (Phages) was unchallenged but treated with phages continuously administrated via drinking water during the first 6 days of the chicks' life (Days 1–6). The third group (*S*E) was not treated with phages but challenged by oral gavage with *Salmonella* Enteritidis LA5 at 5×10^4^ CFU/chick on Day 7. In the fourth group (*S*E + Phages), phages were administrated via drinking water during the first 6 days of the chicks' life (Days 1–6), and chicks were also challenged by oral gavage with *Salmonella* Enteritidis LA5 at 5×10^4^ CFU/chick at Day 7. To check for emerging resistance of *Salmonella* to the phage cocktail, another phage administration was performed 2 days before the end of the trial (on Days 27 and 28) in the “Phages” and “*S*E + Phages” groups. At Days 6, 11, 14, 21, and 28, organs (i.e., gizzard, ileum, cecum, and feces) of 10 chicks per sampling day were collected, weighed, crushed, and homogenized as in trial 1 to quantify *Salmonella* and phages and to identify phages. Chicken cecal contents were also collected and weighed on the same collection days, then frozen at −80°C for subsequent microbiota analysis.

### 2.6 Frequency of bacteriophage‐insensitive *Salmonella* clones

To check for the acquisition of phage resistance, 22 *Salmonella* colonies isolated from plate counts of 5 to 6 cecal contents of phage‐treated birds per sampling days, were tested for susceptibility to each phage of the cocktail. Tests were performed using the spot test assay with 10‐fold dilutions of phages applied on bacterial lawns from each single colony picking. The test establishes whether isolates are as susceptible to phages as the original *Salmonella* Enteritidis LA5 strain, have reduced susceptibility, or are resistant to phages without plaque formation after exposure to the phages in vivo.

## 3 MICROBIOTA ANALYSIS

### 3.1 DNA extraction and 16S rRNA gene sequencing

DNA of the 200 samples from chick cecal contents was extracted using the NucleoMag® DNA Microbiome kit following the manufacturer's instructions (Macherey‐Nagel) and automated via the Microlab Nimbus 100 robot (NIMBUS4 Hamilton). The mechanical lysis step was added with the Precellys Evolution instrument with activated Cryolys (Bertin Technologies) for 6×90 s at 9000 rpm and 4°C. Nanodrop One (Thermo Scientific™) was used to quantity DNA samples. Then, the genomic DNA obtained was sequenced on an Illumina MiSeq by Genomer platform (EMBRC France partner) using paired‐end 2×300 bp cycles. The V3‐V4 regions of the bacterial 16S rRNA gene were PCR amplified using the primers forward ^5'^TCGTCGGCAGCGTCAGATGTGTATAAGAGACAG‐[CCTACGGGNGGCWGCAG]^3'^ and reverse ^5'^GTCTCGTGGGCTCGGAGATGTGTATAAGAGACAG‐[GACTACHVGGGTATCTAATCC]^3'^.

### 3.2 16S rRNA gene sequence analysis

Bioinformatic 16S rRNA gene sequence analyses were performed using the FROGS analysis pipeline (Escudie et al., 2018). The read assembly yielded a total of 9,020,525,16S rRNA gene sequences that were grouped in 2,199,332 clusters using Swarm (Mahe et al., 2014), with a distance of 1. After removal of the chimeric reads (detected using VSearch; Rognes et al., 2016), the low‐quality reads and the samples with low coverage (Nreads < 500) sequences, 4,569,206 sequences were kept and clustered into 869 OTUs. Then, the most abundant OTUs were selected by filtering very rare OTUs (relative abundance < 0.005% of all sequences; Bokulich et al., 2013) and those including sequences matching phiX sequence recorded in a specific databank (Escudié et al., 2018). This filtering step resulted in a final number of 866 OTUs. Finally, OTUs were assigned to the lowest taxonomic category using NCBI BLAST and SILVA 16S v138.1 database (Quast et al., 2013).

### 3.3 Statistical analysis

Statistical analysis and data visualizations were performed using R packages. Relative abundance comparisons of predominant genera were performed using the R phyloseq package (v1.40.0; McMurdie & Holmes, 2013). The “plot_heatmap” function of phyloseq was used to assess the relative abundance of the predominant families. α‐Diversity was measured using Chao1 and Shannon indices and computed using the “estimate_richness()” function of phyloseq. ANOVA tests were performed to determine α‐diversity changes between all the groups and *t* tests were used to test for differences in mean α‐diversity between groups. β‐Diversities were computed and visualized using the “ordinate ()” function and the Bray–Curtis index. Permutational multivariate analysis of variance analysis (PERMANOVA, 9999 permutations) was used to compare mean β‐diversity indices among the groups. This was done using the “adonis2()” function of the R package Vegan (Oksanen et al., 2013). A principal component analysis (PCA) was performed using FactoMineR R‐package (Lê et al., 2008) to summarize the distribution of the relative abundances among the genera. Differential abundances of species between groups at the OTU level were assessed using the DESeq. 2 package (v1.36.0) (Love et al., 2014) (*p* values < 0.01 after correction using Benjamini & Hochberg's method). Tests of differential abundances at the genus level were made using Welch's *t* tests. This was done using STAMP v.2.1.3 (Parks et al., 2014).

Statistical differences in means between groups for the numbers of *Salmonella* (CFU/g) and phages (PFU/g) were assessed using Wilcoxon–Mann–Whitney tests.

## 4 RESULTS

### 4.1 Phages can survive in the chicken gut in the absence of *Salmonella*

To use phages as a preventive treatment, it was first verified if they were able to persist and survive along the chick intestinal tract in the absence of their host bacterial strain. The viability and stability of the phages were determined by enumerating and identifying them in the different organs of the chicks.

**Figure 1:**
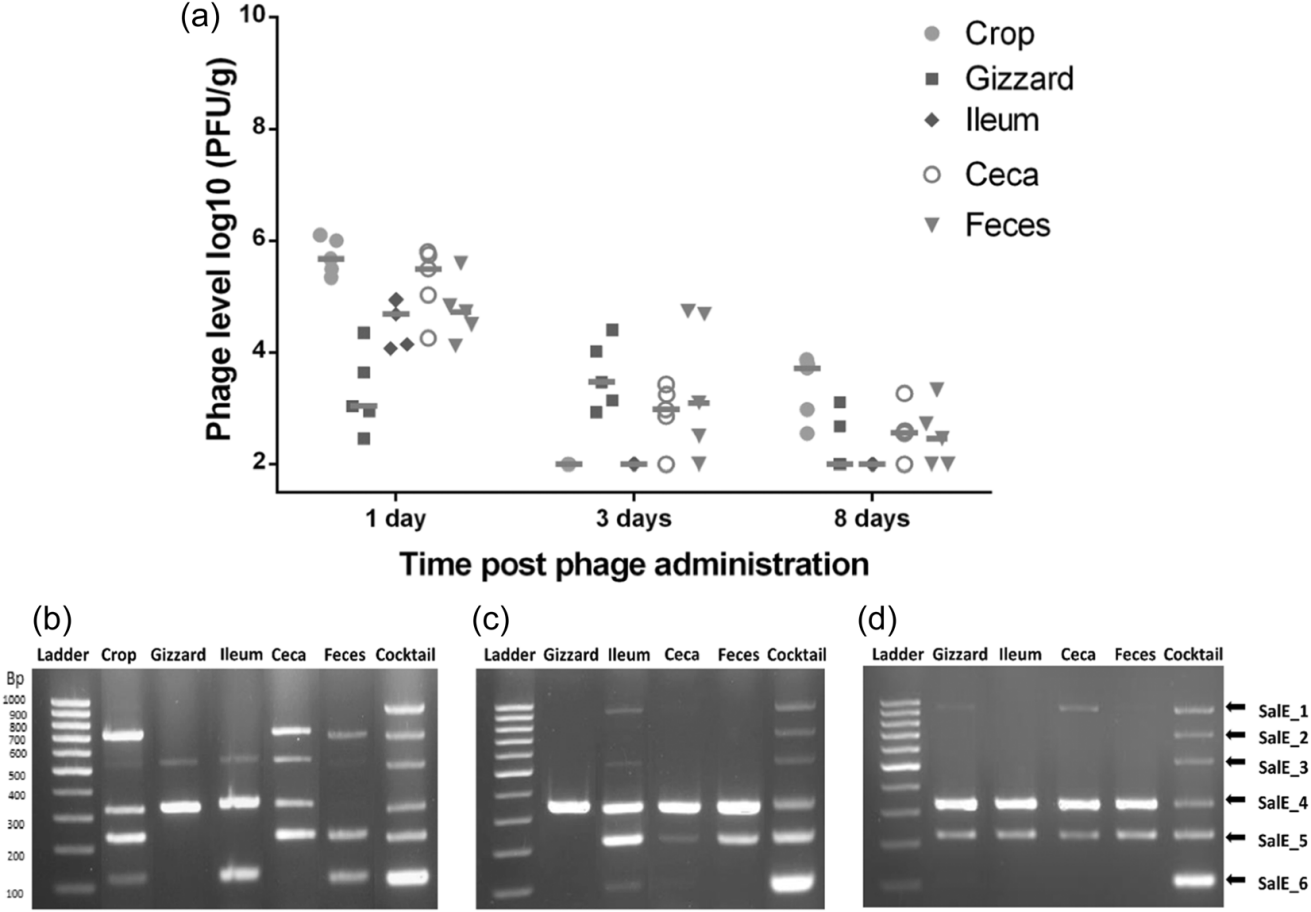
Phage ability to persist and survive in chickens' gut in the absence of their host *Salmonella*. (a) On Days 1, 3, and 8 after oral administration ended, phages were enumerated as PFU/g of the organ from the crop, gizzard, ileum, cecal content, and feces of chicks. Data represent phage amount in each chick's organ after phage administration (5 chicks per time point were tested). The bars represent the median of phage titers. Phages remaining viable in chickens' gut were identified on Days 1 (b), 3 (c), and 8 (d) after phage oral administration ended. Multiplex PCRs were performed with a pool of phages recovered from plate count samples of the crop, gizzard, ileum, cecal content, and feces of chicks.

From Day 7 through Day 14 (1–8 days after phage administration ended), phages were still detected until day 8 in the crop, gizzard, ceca, and feces, though there was a downward trend of the number of phages in all recovered intestinal segments (Figure 1a). On the contrary, phages were not detected in the spleen or liver of chicks. On Day 7, the first day after phage administration was stopped, all chicks were still positive for phages with a titer ranging from 5.81 log10 PFU/g in the crop to 3.76 log10 PFU/g in the gizzard. In the ceca, which is the main site of *Salmonella* colonization, phage concentration was about 5.50 log10 PFU/g with the detection of four phages (SalE_2, SalE_3, SalE_4, and SalE_5) by multiplex PCR (Figure 1a,b). Then, 3–8 days after phage administration ended (from Days 9 to 14 of age), the number of phages in the ceca decreased from 3.07 log10 PFU/g to 2.71 log10 PFU/g with 4 phages found at Day 3 (SalE_1, SalE_4, SalE_5, and SalE_6), and 3 phages from the cocktail (SalE_1, SalE_4, and SalE_5) being detectable at Day 8 (Figure 1c, d).

These data showed that after an oral administration via drinking water, the cocktail phages were able to overcome the pH barrier, persist, and remain viable in the chick intestinal tract for several days in the absence of their *Salmonella* host. It was thus relevant to assess their potential protective activity against *Salmonella* infection.

### 4.2 Prophylactic administration of phages reduces chicken gut colonization by *Salmonella*

**Figure 2:**
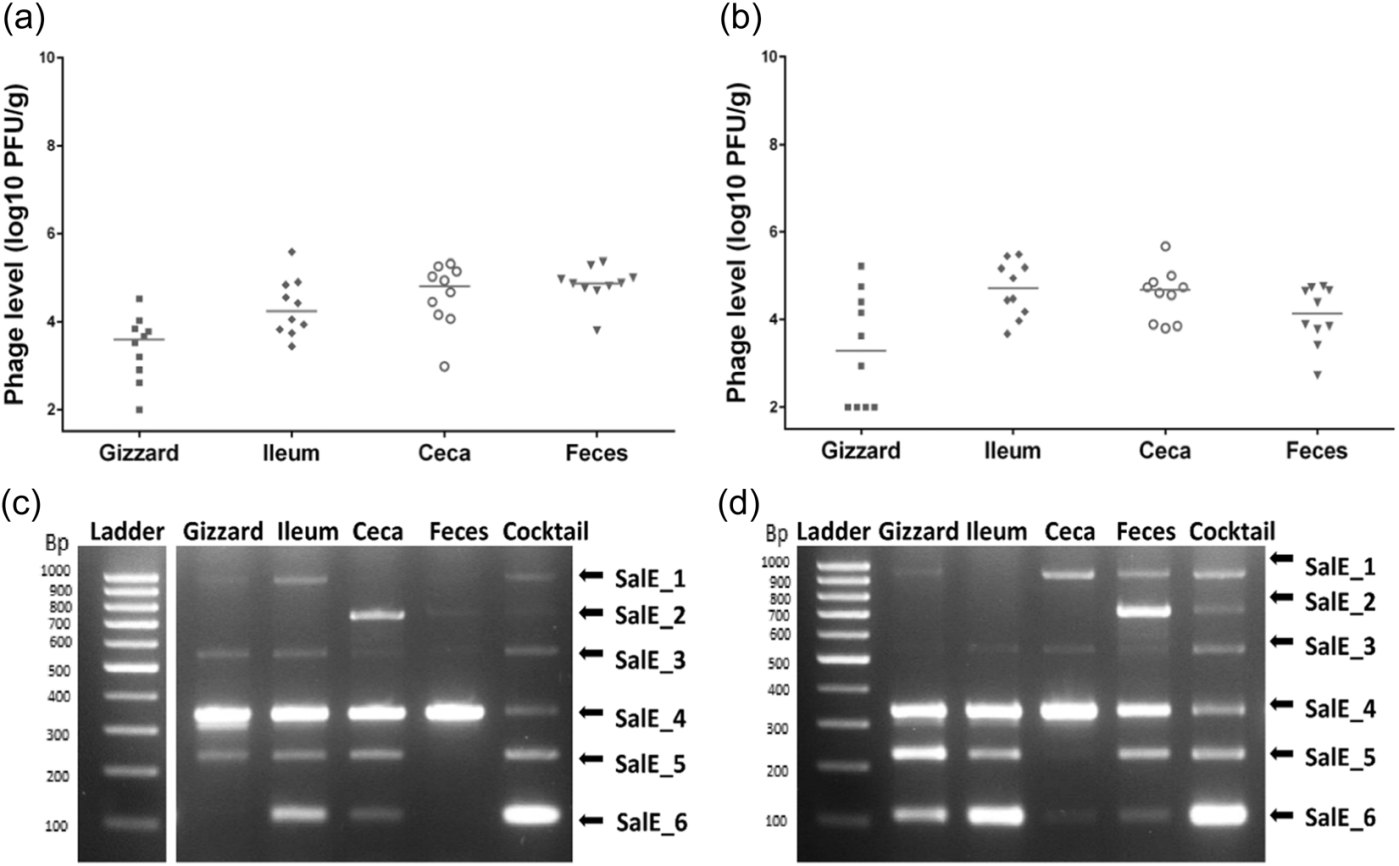
Presence of phages in chicks' gut before the challenge by *Salmonella* Enteritidis (*S*E) at 6 days of age. (a) Phages were enumerated as PFU/g of organs in the gizzard, ileum, ceca, and feces of 10 chicks in the phage‐treated group (“*S*E+Phages”), and (b) in the group treated only with phages (“Phages”). Each symbol represents an animal and the bars represent the median of phage titers. (c) Phages were identified by multiplex PCRs with a pool of phages recovered from plate count samples of each chick's organs in the “*S*E+Phages” group and (d) in the “Phages” group.

To evaluate whether the phage cocktail would be effective in preventing *Salmonella* infection, the *Salmonella* load of the “*S*E” group challenged by *Salmonella* Enteritidis LA5 on Day 7, was compared to the “*S*E+ Phages” group challenged by *S*. Enteritidis LA5 on Day 7, after the phage administration. Before the challenge, no *Salmonella* were recovered in either group, as expected. At 6 days of age, after phage administration was stopped but before *Salmonella* infection, phages were enumerated and identified in the different gut segments of chicks collected from the “Phages” group and “*S*E+ Phages” group that had received phages (Figure 2). The number of phages was on average 4.1 ± 0.82 log10 PFU/g in organ samples from both groups (Figure 2a,b). It is worth noting that all the phages composing the cocktail were identified by the multiplex PCR indicating that phages were present in the chick gut before infection, especially in the ceca. However, it should be noted that varying degrees of PCR signals in the chick gut between the two groups were observed (Figure 2c,d).

**Figure 3:**
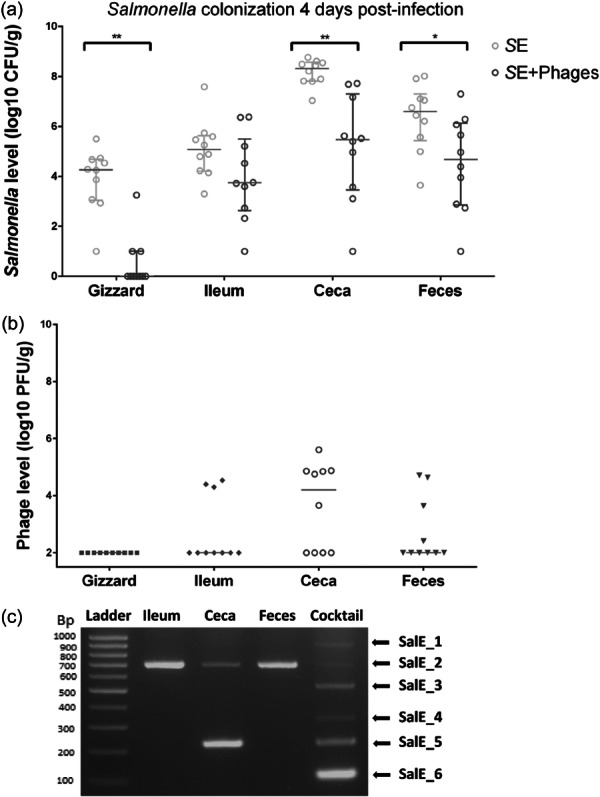
Effect of the prophylactic administration of phages (10^9^ PFU/mL) on *Salmonella* Enteritidis (*S*E) colonization in broiler chickens at 11 days of age (4 days PI). (a) *Salmonella* count was monitored from the gizzard, ileum, cecal content and the feces collected from the group challenged by 5 × 10^4^ CFU/chick at 7 days of age (“*S*E”) represented by gray circles for each animal and from the group that received phages before being challenged (“*S*E+Phages”) represented by black circles for each animal. *Salmonella* was enumerated as CFU per gram of organs in 10 chicks per group. The bars represent the median of *Salmonella* levels. (b) Phage persistence and survival in the gut after the challenge were quantified (“*S*E +Phages group”) as PFU/g of organs. Each symbol represents an animal. The bars represent the median of phage levels. (c) Phages were identified by a multiplex PCR with a pool of phages recovered from plate count samples of each chick's organs. Statistical differences between groups for *Salmonella* counting were calculated using the Wilcoxon test with **p < 0.01 and *p < 0.05.

Preventive treatment with phages for 6 days before infection resulted in a significant reduction of *Salmonella* burden 4 days postinfection in the chicks' different gut compartments (Figure 3a). Indeed, *Salmonella* count was significantly reduced in the “*S*E+ Phages” group compared with the “*S*E” group, on average by 4.3 log10 (*p* = 0.003) in the gizzard, 2.9 log10 (*p* = 0.004) in the ceca and by 1.9 log10 (*p* = 0.048) in the fecal excretion. *Salmonella* reduction in the ileum was not statistically significant but decreased on average by 1.4 log10.

Meanwhile, phages were enumerated and identified from the different gut sections in the “Phages” and “*S*E+ Phages” groups (Figure 3b,c). In the “*S*E + Phages” group, phages were still detectable 5 days after the phage administration was stopped, that is, 4 days postinfection; The phage SalE_2 was detected in the ileum of 3 chicks and the fecal shedding of 4 chicks, and SalE_2 and SalE_5 phages were detected in the cecal content of 6 chicks (Figure 3b,c). For the other chicks, the level of phages was below the limit of detection (≃ 2 log10 PFU/g). This was also the case for all chicks of the “Phages” group. These results suggest that phages were able to infect and kill the *S*E LA5 strain in vivo and that 2 phages from the cocktail were able to survive and replicate in vivo at detectable levels.

**Figure 4:**
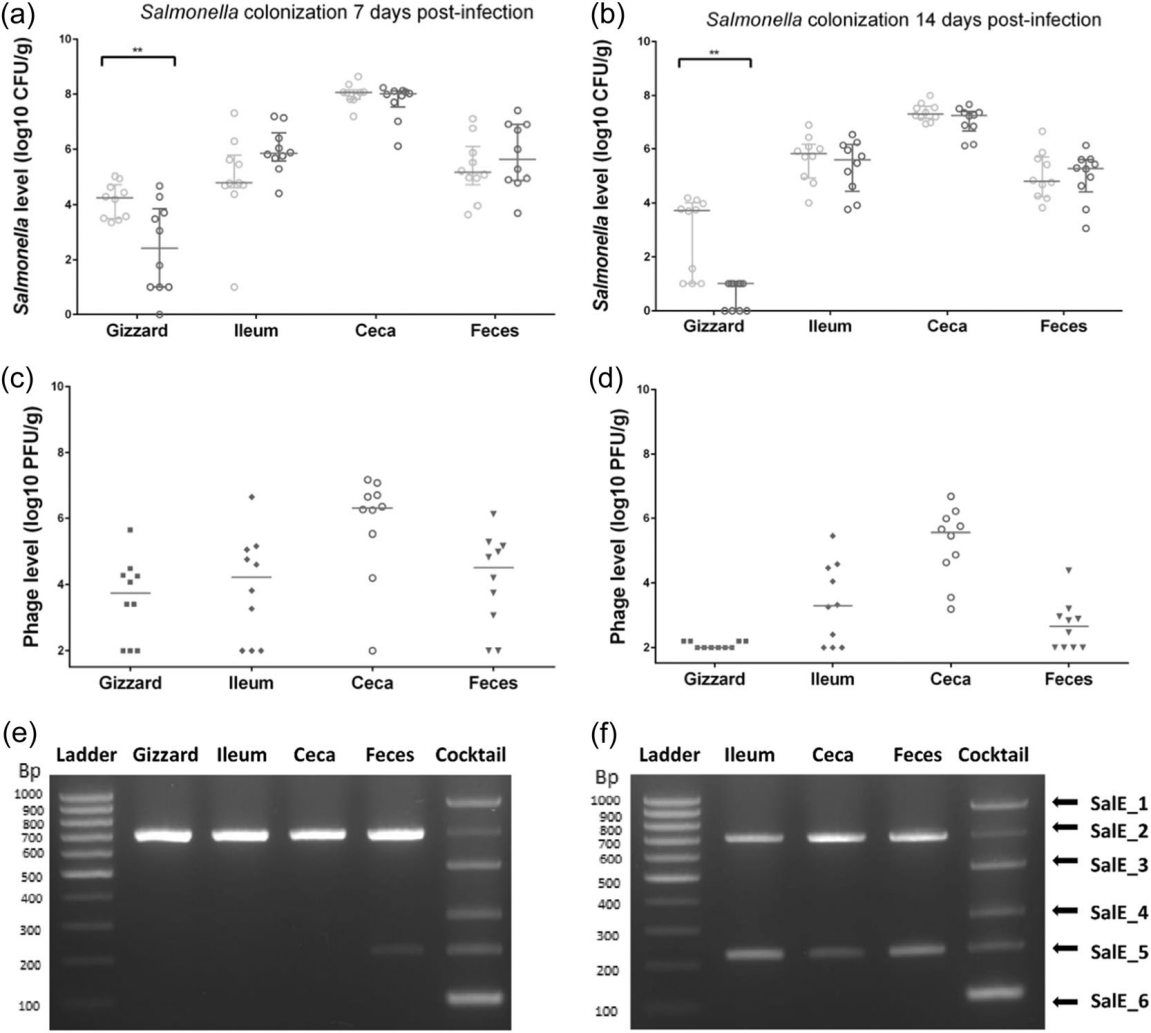
Effect of the prophylactic administration of phages (10^9^ PFU/mL) on *Salmonella* Enteritidis (*S*E) colonization in broiler chickens at 14 and 21 days of age (respectively 7 and 14 days PI). (a) 7 days postinfection and (b) 14 days postinfection, *Salmonella* count was monitored from the gizzard, ileum, cecal content, and the feces collected from the group challenged (“*S*E”) represented by gray circles for each animal and from the group which received phages before being challenged (“*S*E+Phages”) represented by black circles for each animal. *Salmonella* was enumerated as CFU per gram of organs of 10 chicks per group. (c) 7 days postinfection and (d) 14 days postinfection, phage persistence and survival in the gut after the challenge was evaluated (“*S*E+Phages” group) as PFU/g of organs, with each symbol representing an animal. The bars represent the median of phage or *Salmonella* levels. (e) 7 days postinfection and (f) 14 days postinfection, phages were identified by a multiplex PCR with a pool of phages recovered from plate count samples of each chick's organs. Statistical differences between groups for *Salmonella* counting were calculated using the Wilcoxon test with **p < 0.01 and *p < 0.05.

However, *Salmonella* colonization started to recover after 7 days postinfection in the “*S*E + Phages” group, reaching the *Salmonella* colonization level of the “*S*E” group at the end of the experiment, 14 days postinfection (Figure 4). In the gizzard compartment, a significant reduction of 2.1 log10 CFU/g in *Salmonella* load was still noticed (*p* = 0.006). One explanation for the lower number of *Salmonella* in the presence of phages is that the quantity of *Salmonella* in the gizzard is the lowest of the organs tested, with the highest Salmonella:phage MOI. In addition, it is an acid environment, low in microbiota, where the chances of *Salmonella*‐phage encounters are highest. These conditions therefore favor the action of phages, which was already observed at 7 days postinfection. The absence of phages at D14 can be explained by the very low number of *Salmonella*. Surprisingly, phage replication reached 5.6 log10 PFU/g in ceca, as shown in Figure 4c,d. As previously observed, the multiplex PCR showed that the two persistent phages were SalE_2 and SalE_5 (Figure 4e,f).

To understand these different capacities of phages to persist in the presence of *Salmonella* Enteritidis LA5 strain in vivo, phage replication dynamics were investigated in vitro.

### 4.3 The replication dynamics of phages in vitro explain in part their persistence in vivo

**Figure 5:**
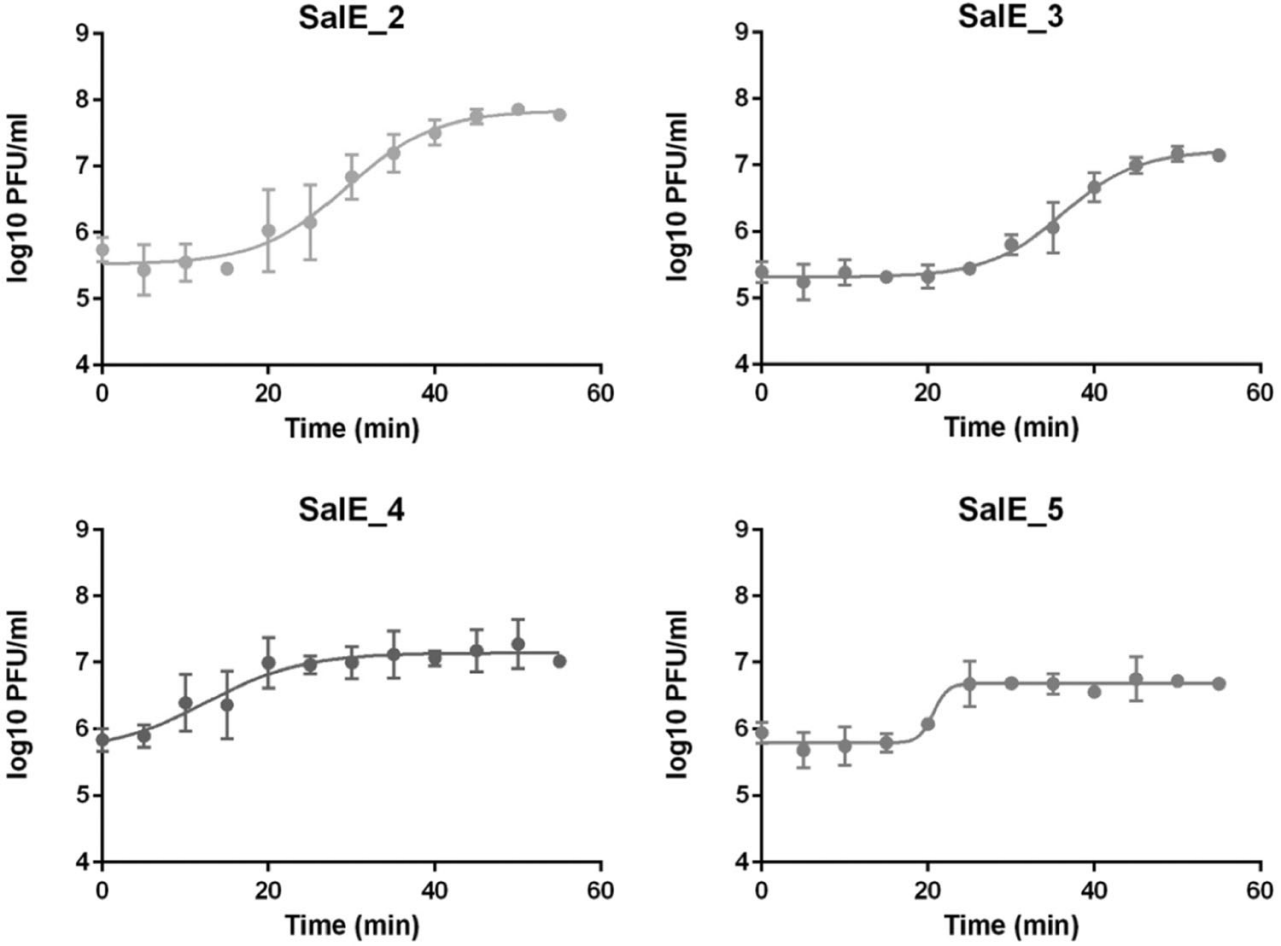
One‐step growth curves of SalE_2, SalE_3, SalE_4 and SalE_5 phages in *Salmonella* Enteritidis (*S*E) LA5 at a multiplicity of infection (MOI) of 0.1. Data represent the mean ± SD of three independent experiments. Phage replication dynamics were evaluated due to their lytic activity on the strain. The phages SalE_1 and SalE_6 which do not replicate within *S*E LA5, were not tested.

To determine the replication dynamics of each phage from the cocktail, one‐step growth curves were performed with *Salmonella* Enteritidis LA5 strain at an MOI (multiplicity of infection) of 0.1 (Figure 5). Phage replication dynamics of SalE_1 and SalE_6 were not evaluated because they do not replicate within the *S*E LA5 strain. The one‐step growth curve for SalE_4 showed a latent period of 5 min while phages SalE_2, SalE_3, and SalE_5 showed a latent period of 15, 20, and 15 min, respectively. Regarding phage proliferation, the one‐step growth curve showed a burst size of 102 PFU/bacterium released for SalE_2 in contrast to the SalE_3 and SalE_4 which had a burst size of 56 and 14 PFU/bacterium released respectively. The phage SalE_5 had a burst size of only 5 PFU/bacterium, which cannot explain its high level in vivo. On the contrary, the phage SalE_2 appears to show rapid infection and proliferation abilities in the *Salmonella* strain which could explain its higher concentration in vivo.

### 4.4 *Salmonella* isolates from the in vivo experiment are still susceptible to most of the phages in the cocktail

**Figure A1:**
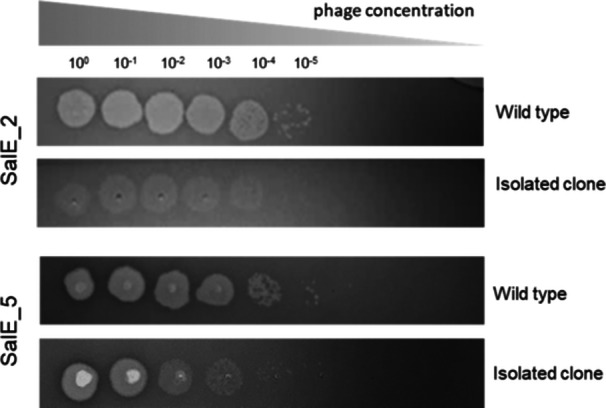
Plaque formation induced by SalE_2 and SalE_5 on lawns of *Salmonella* Enteritidis LA5 (wild‐type strain) and on lawns of selected isolated clones from *Salmonella* plate counts of chicken cecal content. The spot assay was used to test the sensitivity of the isolated clones to phages by spotting serial dilutions (1:10 dilutions) of phages on isolates to compare their plaque formations to those obtained on the wild type.

By the end of the animal trial, Day 14 postinfection, the significant phage effect on *Salmonella* levels in ceca and feces observed 4 days postinfection was no longer detected. To determine whether the reduction of efficiency could be due to the acquisition of bacterial strain resistance toward the cocktail of phages, the phage susceptibility of *Salmonella* strains recovered from the animals was tested. Twenty‐two colonies were randomly isolated from *Salmonella* plate counts of chicken cecal contents throughout the trial. Resistant clones showing a reduction in sensitivity in terms of a reduction in phage titer relative to the phage titer on the wild‐type strain, but also a reduction in sensitivity manifested by the appearance of turbid plaques without any reduction in phage titer, were recorded as “less susceptible.” Of the tested clones, 64% were less susceptible to SalE_2 (14/22 clones) and 27% were less susceptible to SalE_5 (6/22 clones). No isolate was less susceptible or resistant to the other phages (SalE_1, SalE_3, SalE_4 and SalE_6). Among the 14 isolates that were less susceptible to SalE_2, the phage titer itself was not reduced but the lawn of bacteria was not completely lysed by the phage leading to the formation of turbid plaques (Figure A1). Conversely, reduced phage titer was observed with the phage SalE_5 on the six less susceptible isolates. The emergence of less susceptible isolates among the surviving *Salmonella* clones was only observed with the two phages that persisted in the trial but not against the other phages.

### 4.5 An additional phage administration before slaughter may further alleviate the *Salmonella* burden in vivo

**Figure 6:**
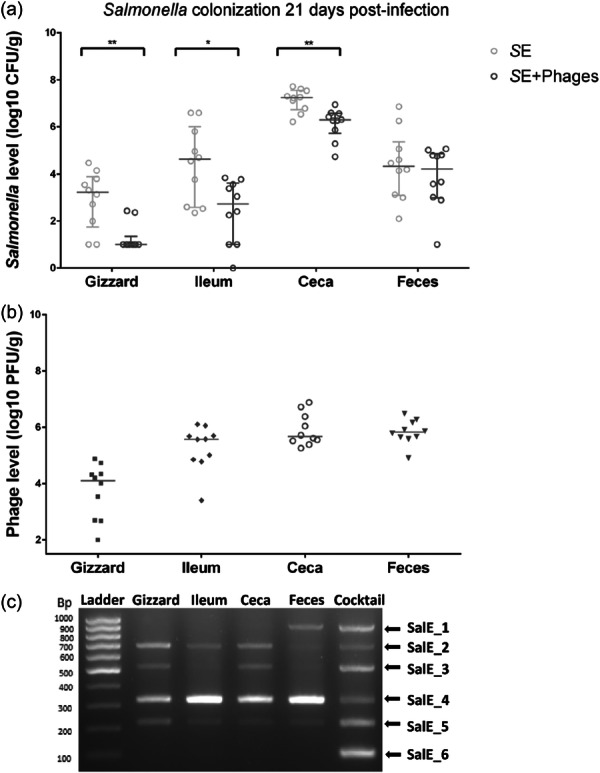
Impact of an additional administration of phages (10^9^ PFU/mL) on the two last days of the trial (27 and 28 days of age) on *Salmonella* Enteritidis (*S*E) colonization in broiler chickens at 28 days of age (21 days PI). (a) *Salmonella* count was monitored from the gizzard, ileum, and cecal content and the feces collected from the group challenged by 10^4^ CFU/chicks at 7 days of age (“*S*E”) represented by gray circles for each animal and from the group that received phages before being challenged (“*S*E+Phages”) represented by black circles for each animal. *Salmonella* was enumerated as CFU per gram of organs of 10 chicks per group. The bars represent the median of *Salmonella* levels. (b) Phage persistence and survival in the gut after the challenge were evaluated (“*S*E+Phages” group). Phages were enumerated as PFU/g of organs, with each symbol representing an animal. The bars represent the median of phage levels. (c) Phages were identified by a multiplex PCR with a pool of phages recovered from plate count samples of each chick's organs. Statistical differences between groups for *Salmonella* counting were calculated using the Wilcoxon test with **p < 0.01 and *p < 0.05.

To further determine whether *Salmonella* at the end of the trial was still susceptible or resistant to the phage cocktail, an additional dose of phages was administrated to chickens via the drinking water on the last 2 days of the trial (at 27 and 28 days of age) in the “*S*E+ Phages” group that had been previously treated with phages. Figure 6a shows that additional phage administration significantly reduced *Salmonella* in the “*S*E+ Phages” group, compared to the “*S*E” group, by 1.7 log10 (*p* = 0.004) in the gizzard, 1.8 log10 (*p* = 0.013) in the ileum and 1 log10 (*p* = 0.01) in ceca, 21 days postinfection. *Salmonella* fecal shedding was not significantly reduced. Phage concentration ranged from 4.2 to 5.8 log10 PFU/g in the different gut segments and, as expected due to the oral inoculation of the cocktail phages just before sampling, 5 out of 6 phages composing the cocktail were detected in all gut sections (Figure 6b,c). This data further suggests that *Salmonella* did not become resistant to all of the phages composing the cocktail and that these phages could be used not only as a preventive treatment but also to treat the ongoing chicken infection.

### 4.6 The phage preventive treatment has no deleterious effect on cecal microbiota

To determine whether prophylactic administration of the phage cocktail to chicks would impact chicken's gut microbiota, the cecal microbiota of chickens in each group was characterized by 16S rRNA gene sequencing at 6, 11, 14, 21, and 28 days of age.

**Figure 7:**
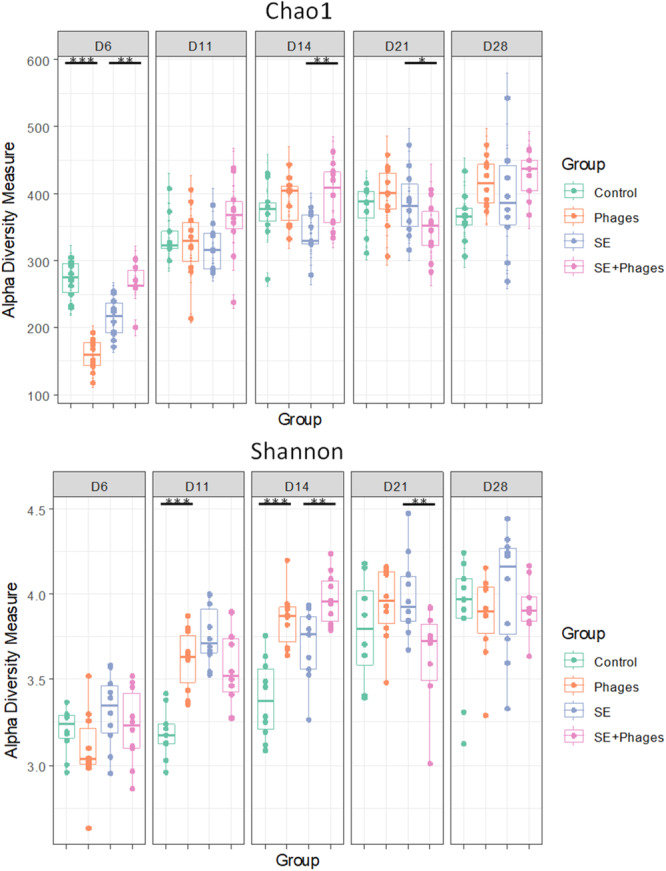
Alpha diversity analysis during the trial at 6, 11, 14, 21, and 28 days of age. Bacterial diversity using Chao1 and Shannon metrics is represented by boxplots to compare the microbiota of chicken cecal content from the "Control” group, the “Phages” group, the “*S*E” group, and the “*S*E+Phages” group. Bacterial 16S rDNA gene content of 10 ceca of chicks per group and per day of age were processed by metabarcoding analysis. Statistical differences between the “Control” and “Phages” groups and between “*S*E” and “*S*E+Phages” groups are highlighted on the graph by using *t*‐tests with *p < 0.05, **p < 0.01, ***p < 0.001.

Changes in microbial α‐diversity analysis over time were assessed using a comparison of the two metrics Chao1 and Shannon. At 6 days of age (before infection), diversity estimated by the Chao1 index was significantly different between the “Control” and the “Phages” groups, and between the groups that will be infected, “*S*E” and “*S*E+ Phages” (Figure 7). Since no significant differences were observed with the Shannon equitability index, one may suggest that significant differences observed at 6 days of age in α‐diversity did not rely on predominant taxa. The value of α‐diversity indices over time increased with chick age in all groups (Figure 7). This may correspond to the maturation of the microbiota over time during chickens' growth. Besides this increase, the α‐diversity tended to stabilize and homogenize between groups over time. Indeed, after 6 days of age, no differences were noticed between the “Control” and the “Phages” groups in diversity estimated by the Chao1 index, as well as the diversity estimated by the Shannon index from 21 days of age. On the last day of the trial, no significant differences were observed in microbial α‐diversity between groups, with any of the indexes under scrutiny.

**Figure 8:**
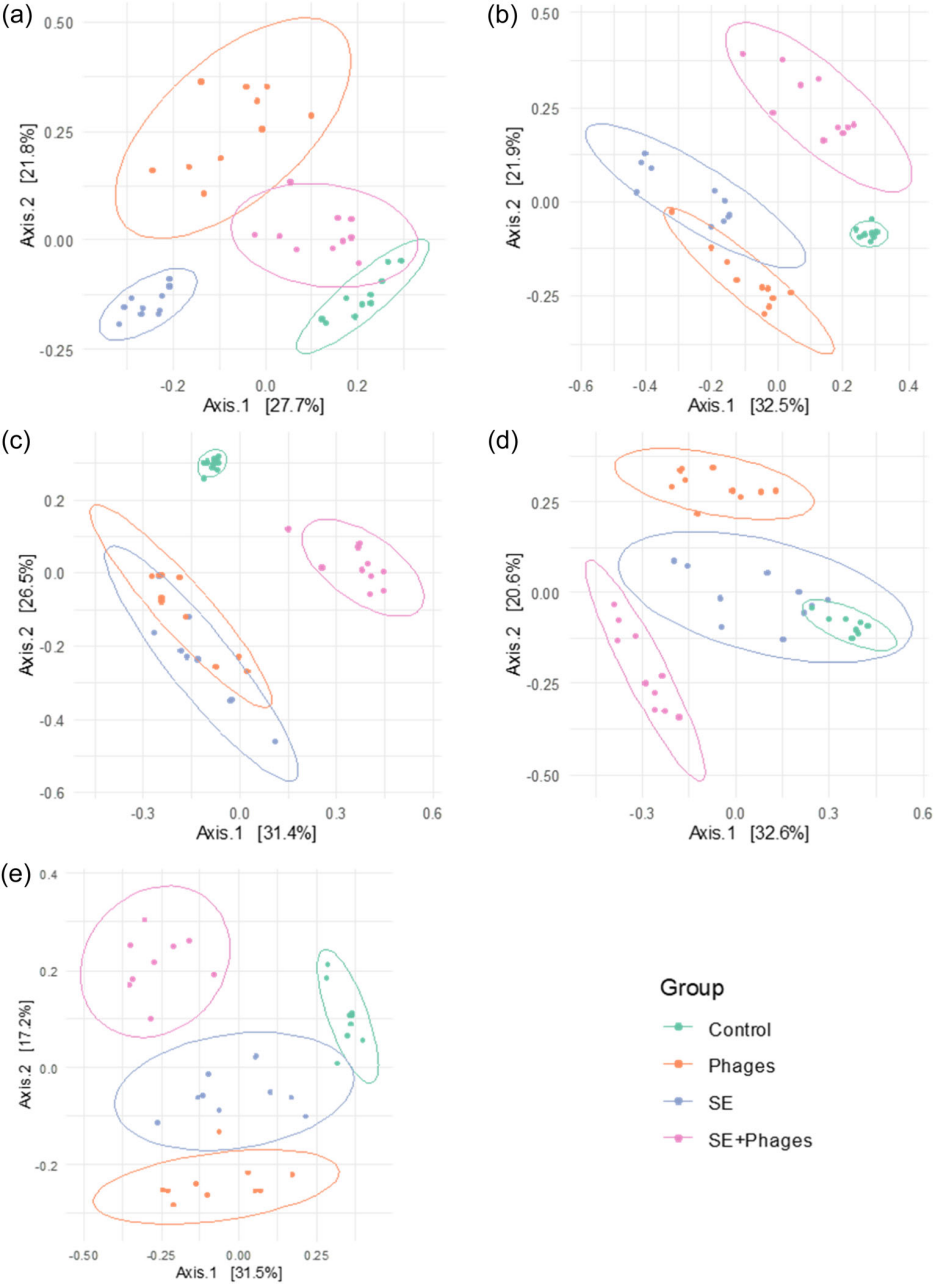
Beta diversity analysis based on Bray–Curtis distances representing dissimilarities between “Control,” “Phages,” “*S*E” and “*S*E+Phage” groups. Each figure panel corresponds to a sampling time at (a) 6, (b) 11, (c) 14, (d) 21, and (e) 28 days of age and is plotted by Multidimensional Scaling (MDS). A single point represents a sample collected from a chicken cecal content (*n* = 10 per group) that is colored by group and clustered in ellipses. Statistical analysis was performed using PERMANOVA tests between each pair of groups and showed that all pairs of groups were significantly different (***p < 0.001).

Despite α‐diversity homogenization over time, β‐diversity analysis estimated by Bray–Curtis distances highlights dissimilarities between all groups since Day 6 of age (Figure 8). Indeed, at 6 days of age, the microbial composition of each pair of groups was significantly different (*p* < 0.001, PERMANOVA test) and these dissimilarities persisted over time. It is worth noting that significant differences were also observed between the two groups strictly subjected to the same conditions at 6 days of age namely the “Phages” and “SE+ Phages” groups and between the “Control” and “*S*E” groups. Each group appears therefore to have developed its own microbiota composition initially determined by the environment or/and the treatment.

**Figure 9:**
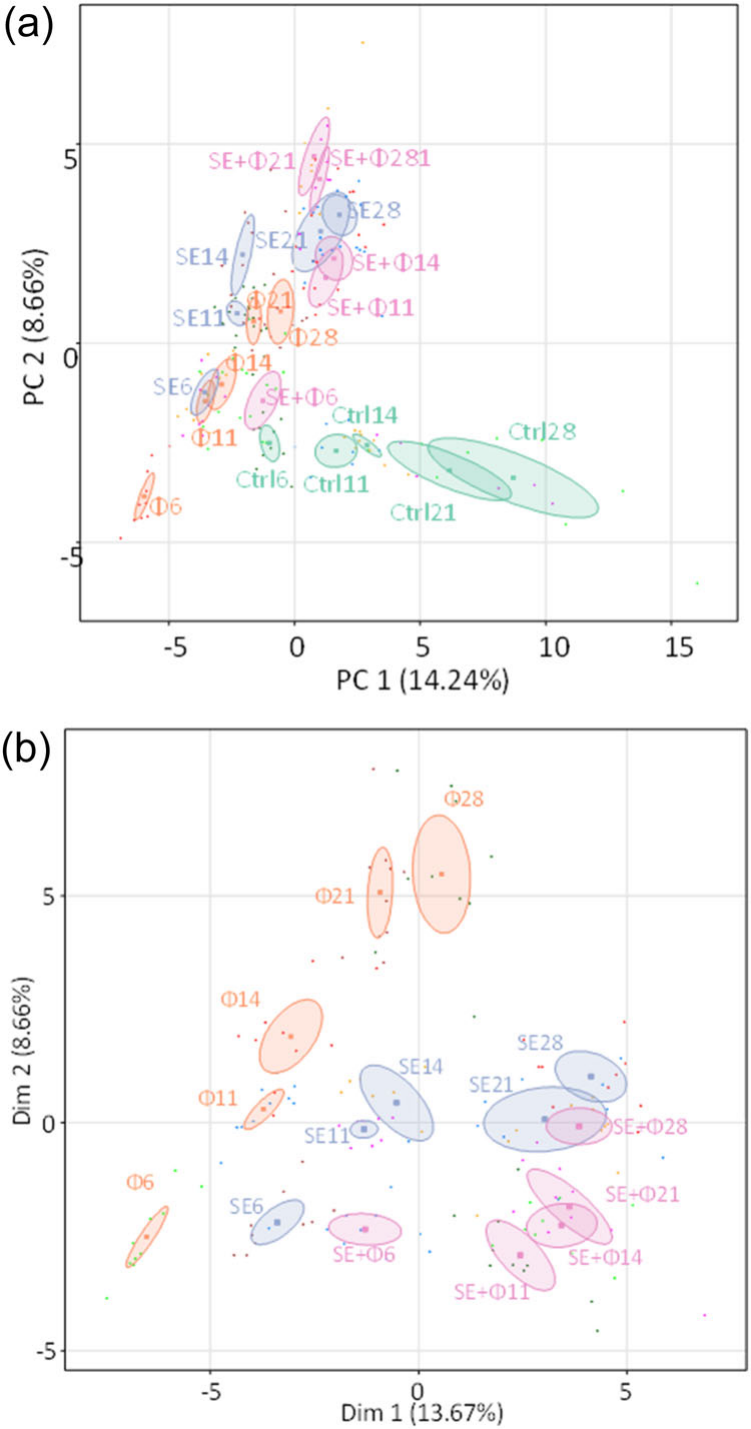
Principal component analysis (PCA) summarizing the distribution of genus abundances. (a) The PCA was based on the cecal bacterial community in the “Control” (Ctrl), “Phages” (Φ), “*S*E” and “*S*E+Phages” (*S*E + Φ) groups (b) with a focus on the 3 groups “Phages” (Φ), “*S*E” and “*S*E+Phages” (*S*E + Φ) groups. Ellipses of the data obtained at the different time points (on Days 6, 11, 14, 21, and 28) are represented in different colors according to the group.

A principal component analysis (PCA) was performed at the genus level to summarize bacterial community changes over time. It also revealed a distinct composition of the gut microbiota of the groups from Day 6 of the trial (Figure 9). The PC1 is positively correlated to *Negativibacillus* and *Eggerthella* genus and negatively correlated to *Enterobacter* and the PC2 is positively correlated to *Shuttleworthia* and *Faecalibacterium* genus and negatively correlated to *Lactobacillus*. The PCA shows the evolution of microbiota tending to diverge over time in the 4 groups and especially between the “Control” and the other groups (Figure 9a). The PCA represented in Figure 9b only analyzes the distribution of genus abundances in the three groups that received treatment (i.e., by excluding the control group). These plots support the hypothesis, that both phage treatment and *Salmonella* modulate microbiota composition.

**Figure A2:**
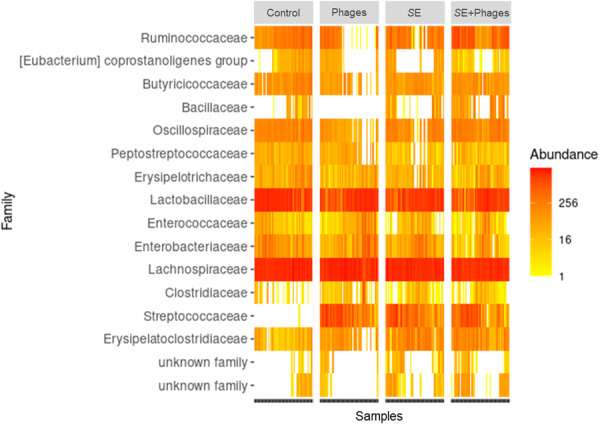
Heatmap showing the relative abundance of the top most abundant families from chicken cecal samples collected throughout the experiment from the “Control,” “Phages,” “*S*E,” and “*S*E+Phages” groups. Each color bar represents the relative abundance of one sample (*n* = 50 per group; i.e., 10 chicks at the five time points). The abundance levels are represented using a gradient of colors. The data are sorted by days of age within a group.

Differences in microbial composition that explain dissimilarities between groups were then explored. This analysis revealed that, at the family level, the groups shared the same 10 most abundant bacterial families. These included *Lachnospiraceae*, *Lactobacillaceae*, *Streptococcaceae*, and *Ruminococcaceae*, which were predominant, followed by *Butyricicoccacea*, *Enterobacteriaceae*, *Enterococcaceae*, *Erysipelatoclostridiaceae*, *Erysipelatotrichaceae*, and *Oscillospiraceae* families (Figure A2). An exception is displayed by the Control group, where the *Streptococcaceae* family is not present.

**Figure 10:**
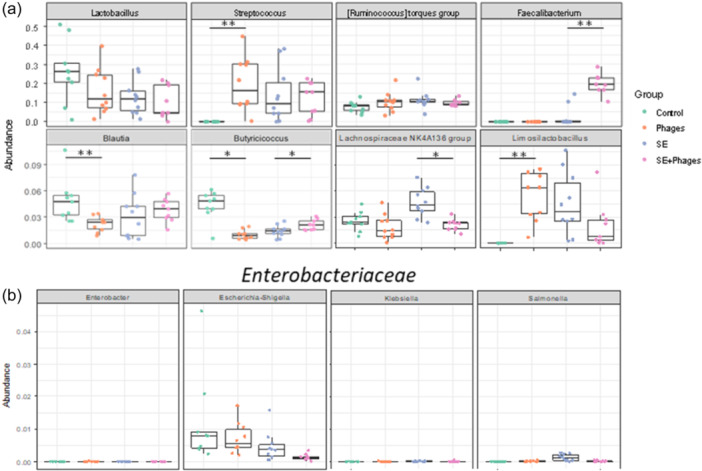
Relative abundance of the most abundant genera in the chicken cecal content of 28‐day‐old chickens. (a) The most abundant genera present in the different groups “Control,” “Phages,” “*S*E,” and “*S*E+Phages” are represented. (b) The most abundant genera of the *Enterobacteriaceae* family in the different groups are represented. Boxplots represent the relative abundance of each sample in each group (*n* = 10 per group). The graph only highlights the comparison of the relative abundances of genera in the “Control” group to those in the “Phages” group and those in the “*S*E” group to those in the “*S*E+Phages” group. Statistical differences in abundances between groups were calculated using Welch's *t* tests with corrected *p* values. *p < 0.05, **p < 0.01, ***p < 0.001.

A statistical analysis of the relative abundance of the most abundant genera was performed (Figure 10). At 28 days of age, the relative abundances of *Limosilactobacillus* and *Streptococcus* from the “Phages” group were found to be significantly higher than those of the “Control” group (*p* = 0.006 and *p* = 0.005 respectively) and *Blautia* relative abundance was significantly lower in the “Phages” group (*p* = 0.008). Comparing the relative abundances of genera in the infected groups, *Faecalibacterium* in the “*S*E+ Phages” group was significantly higher (*p* = 0.009) than in the “*S*E” group. As the phage cocktail targets *Salmonella* species, a focus was made on genera of the *Enterobacteriaceae* family. As expected, *Escherichia‐Shigella* and *Salmonella* abundances were reduced in the “*S*E+ Phages” group compared to the “*S*E” group (*p* = 0.01). *Escherichia‐Shigella* genus tends to decrease also in the “Phages” group versus the “Control” suggesting that phage cocktail can target these bacteria.

**Figure 11:**
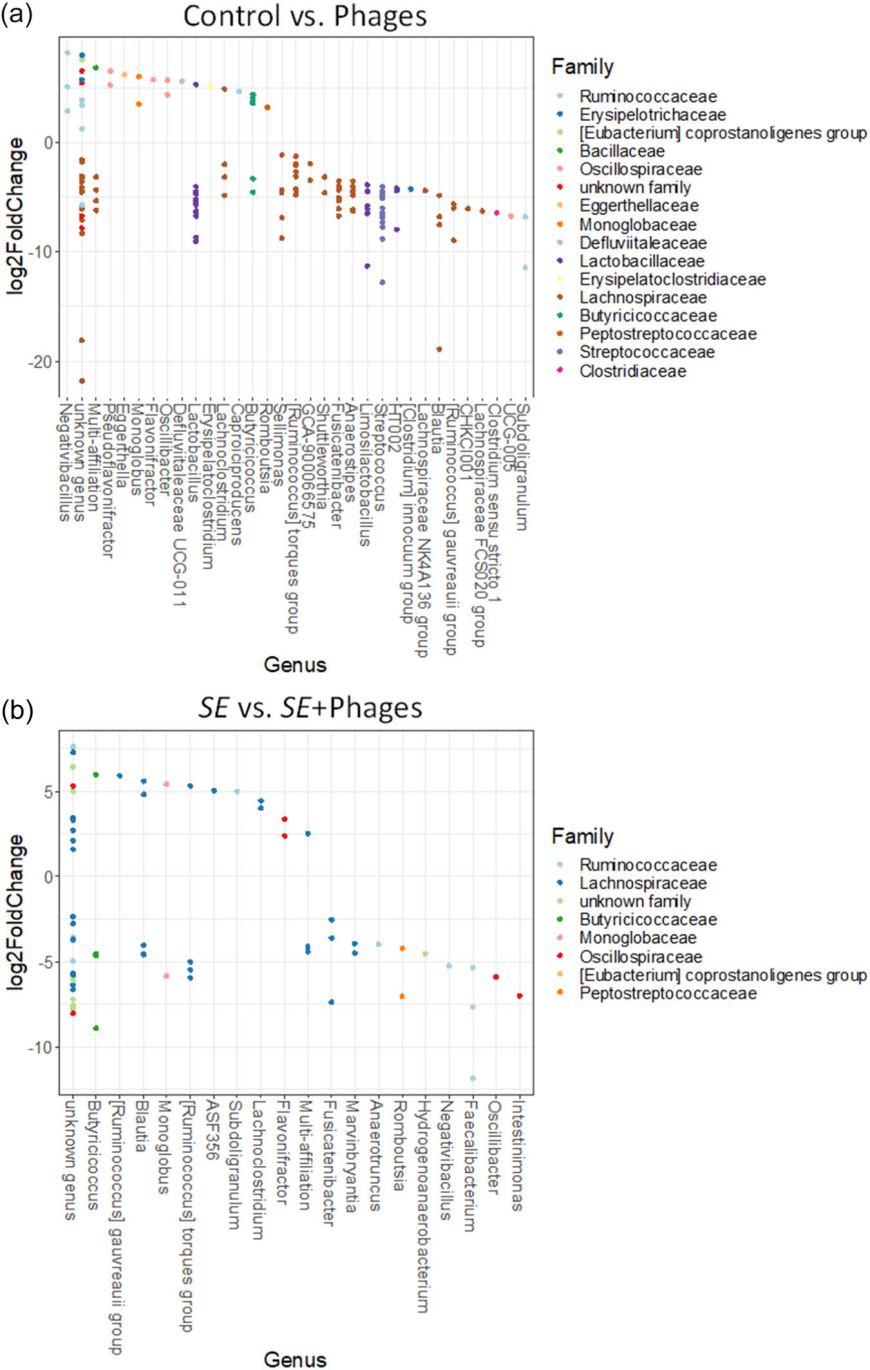
Differential abundance analysis using DESeq2 displaying the log2‐fold changes in genus abundance at 28 days of age. (a) Comparison between the “Control” versus “Phages” group and (b) between the “*S*E” versus “*S*E+Phages” group. 141 OTUs were differentially abundant (16%) in (a) and 73 OTUs were differentially abundant (8%) in (b). Each single point represents an OTU ranked by the corresponding genus and colored according to the family to which it belongs. A negative Log2‐fold change represents genera enhanced in the “Phages” or “*S*E+Phages” groups, while a positive Log2‐fold change represents genera enhanced in the “Control” or “*S*E” groups.

Lastly, changes at the OTU level were explored by differential abundance analysis in groups treated with phages versus groups not treated (Figure 11). OTUs belonging to the genera such as *Subdoligranulum*, *Blautia*, *Streptococcus, Limosilactobacillus*, *Lactobacillus*, and *Fusicatenibater* were enriched in the “Phages” group versus “Control” group. Genera such as *Faecalibacterium*, *Rombutsia*, *Fusicatenibacter*, and *Lactobacillus* were enriched in the “*S*E+ Phages” group versus the “*S*E” group. Furthermore, in both phage‐treated groups (“Phages” and “*S*E+Phages”) genera *Lactobacillus* and *Fusicatenibater* were enriched and genus *Flavonifractor* was depleted, suggesting that they might be impacted by phage treatment.

**Figure A3:**
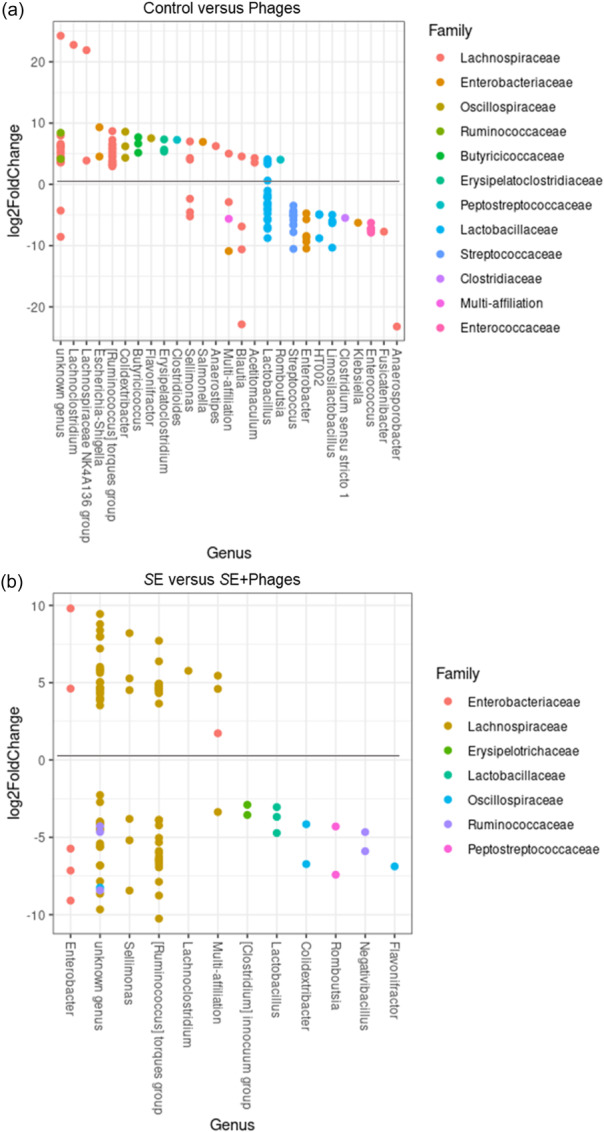
Differential abundant analysis using DESeq2 displaying the log2‐fold changes in genus abundance at 6 days of age between (a) the “Control” versus “Phages” group and (b) the “*S*E” versus “*S*E+Phages” group. 127 OTUs were differentially abundant (14%) in (a) and 104 OTUs were differentially abundant (12%) in (b). Each single point represents an OTU ranked by the corresponding genus and colored according to the family to which it belongs.

**Figure A4:**
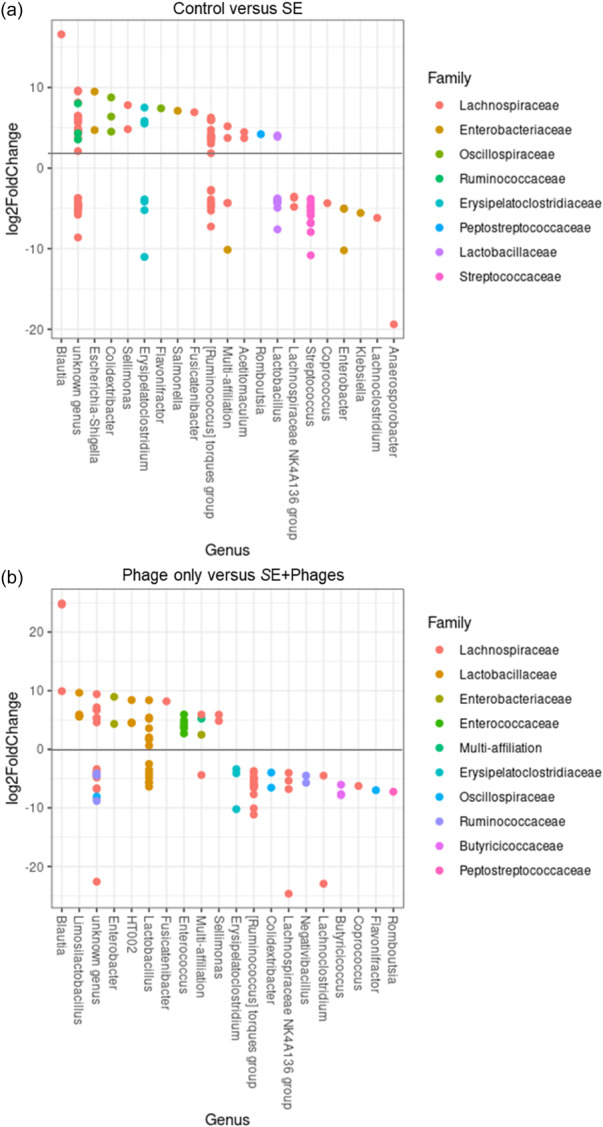
Differential abundant analysis using DESeq2 displaying the log2‐fold changes in genus abundance between groups raised in similar conditions at 6 days of age: (a) The “Control” versus “*S*E” group and (b) the “Phages” versus “*S*E+Phages” group. 117 OTUs were differentially abundant (13%) in (a) and 97 OTUs were differentially abundant (11%) in (b). Each single point represents an OTU ranked by the corresponding genus and colored according to the family to which it belongs.

From 6 days of age, some genera were already enriched in “Phages” group versus “Control,” namely, *Fusicatenibacter*, *Limosilactobacillus*, *Streptococcus*, and *Lactobacillus* as well as *Rombutsia* and *Lactobacillus* enriched in “*S*E+ Phages” group versus “*S*E” group (Figure A3). However, some differences in abundance were already present initially between groups, independently of treatments. This is the case of OTUs of the genus *Streptococcus*, as confirmed by the analysis of differentially abundant OTUs between groups subjected to similar conditions at 6 days of age (Figure A4). These results are consistent with the influence of the environment on the gut microbiota. This influence may explain why the gut microbial composition tended to diverge at the beginning of the experiment and why these differences may persist over time. Other differences in abundance between groups are detected for OTUs that were not present on the first days (Figures A3 and A4) and that emerged over time (Figures 10 and 11), as is the case for OTUs of the genus *Faecalibacterium*. This type of result suggests potential phage effects on certain taxa, whether or not jointly with the environmental effect. Nevertheless, it is noteworthy that, despite the potential modulatory effect of phages on the microbial community, the potential effect of phages did not include taxa known to have a deleterious effect on the gut microbiota or the host.

## 5 DISCUSSION

In the present study, we highlight the potential of a prophylactic phage treatment in chicks during their first days of life to prevent the development of infection by *Salmonella* Enteritidis. The early growth stage of chicken is a critical period for *Salmonella* implantation due to the immaturity of their immune system and microbiota and thus, a key period for the control of that pathogen (Ballou et al., 2016; Crhanova et al., 2011; Litvak et al., 2019). Most phage‐based treatments against *Salmonella* have focused on their therapeutic use by treating ongoing infection to cure the disease in chicken (Atterbury et al., 2007; Fiorentin et al., 2005; Gonçalves et al., 2014; Lim et al., 2012) but few have investigated the potential for prophylactic use of phages in chicks to prevent the establishment of infection (Ahmadi et al., 2016). Despite *Salmonella* reduction in many of these studies, the therapeutic strategy may be limited by significant economic losses in parallel as it may involve the treatment of advanced infections that require more extensive measures or large culling.

One of the biggest challenges to effective bacteriophage prophylaxis in animals as well as for therapy, is the survival and stability of bacteriophages that are orally administered in the absence or the presence of the target bacteria. Phages must remain viable and abundant in the intestine to be available for future and ongoing infection. These parameters are important and will determine phage efficacy in vivo (Khan & Rahman, 2022; Li et al., 2022; Ly‐Chatain, 2014). In our study, we reported that in the trial without infection and on Day 6 (1 day before the infection step) of the 2nd trial, the phages were viable in the gut after oral consumption in the absence of their host bacteria. On the latter one, approximately 5.5 ± 0.4 log10 PFU/g were found in the cecal contents of chicks without coadministration of antacids, which is often used in phage therapy (Atterbury et al., 2007; Colom et al., 2017; Loc Carrillo et al., 2005; Richards, Connerton et al., 2019), suggesting that they were able to survive a low acidic environment across the gastric part of the chicken gut. Furthermore, *Salmonella* reduction 4 days postinfection further suggests that the phages persisting in the gut were also active and able to kill *Salmonella*.

These results showed that preventive application of phages significantly reduced *Salmonella* burden by 3 log10 CFU in chicken guts for at least 4 days after the infection. These encouraging results are in concordance with another study which showed that prophylactic phage treatment prevents the establishment of *S*. Enteritidis infection in quail and demonstrated that it could reduce the *S*. Enteritidis colonization more effectively than phage administration post‐challenge (Ahmadi et al., 2016). In two other animal models, reductions similar to those in our study have been observed. *Salmonella* colonization was significantly decreased for at least 4 and 5 days postinfection, respectively, in mice and pig models (Lamy‐Besnier et al., 2021; Thanki et al., 2022). In another study where phages were administrated continuously (prophylactically and therapeutically) through to mash diet, phages also showed to be effective in reducing *Salmonella* colonization in chickens (Thanki et al., 2023). However, our treatment did not exhibit a long‐term effect. We can speculate, nevertheless, that a lower infectious dose of *Salmonella* (≤10^3^ CFU), more in line with infection in field conditions, would lead to a more efficient treatment and complete protection toward *Salmonella*. It has been described, indeed, that as few as 10–100 bacteria can cause infection in young chicks (Humphrey, 1999).

Resurgence of bacteria was observed at the end of the trial due to their incomplete elimination from chickens' gut. Phage disappearance from the gut and phage resistance appear to be the two main hypotheses that could explain this observation.

As it is commonly found, phages have relatively short resident times with rapid clearance from the digestive tract (Khan & Rahman, 2022; Ly‐Chatain, 2014). This phenomenon leads to in vivo reduction in phage titer and could affect phage treatment effectiveness. It is therefore important to ensure a significant level of various phages from a cocktail is available and therapeutically effective during the pathogen infection. From 4 days PI (postinfection) to 14 days PI, two phages of the cocktail (with SalE_2 prevalence) persisted in lower abundance (4 log10 PFU/g) while the bacteria amplified, resulting in a progressive decline of the MOI. The lack of effectiveness after 4 days PI may be explained by the disappearance of the other phages of the cocktail, and thus, by the lower variety of phages present to tackle *Salmonella*. This appears to be the main cause of the reduced efficacy. As phage counting on the *S*. Newport strain (sensitive to the phage cocktail except for SalE_2) revealed few if any plaque formations, whereas phage counting on *S*. Enteritidis (sensitive to SalE_2) revealed a high number of plaque formations, only SalE_2 appeared to replicate. One reason may be that this phage showed stronger amplification on the challenge strain with a relatively short latent period of 15 min and a high burst size of 102 PFU released per bacteria. The overabundance of a single phage could have enhanced the risk of phage‐resistant bacteria emerging, which would be consistent with the second hypothesis. Indeed, the presence of a single phage is more likely to induce resistance than the use of a phage cocktail.

As summarized in (Oechslin, 2018), several studies have reported the development of phage resistance during phage therapy in vivo. Our results showed that the relatively short administration of phages for 6 days, did not induce resistance to the cocktail during the 4 days following infection. However, from 7 days PI, the emergence of phage resistance to the persistent phage SalE_2 was observed along with the increase of *Salmonella* concentration. The recovered isolates were less susceptible to the single persistent phage but were still susceptible to the less persistent phages in the cocktail. This result confirms that the phage resistance that occurred was more the result of the exposure of the bacteria to a single persistent phage. Consistent with this suggestion was the significant reduction in the *S*. Enteritidis concentration achieved after the resumption of phage administration during the ongoing infection. The presence of only 2 phages and the absence of other phages affected the long‐term efficacy of the phage cocktail. The persistence of the other phages, using a different receptor, could compensate for the reduction in efficacy due to resistance to a single phage, and prolong the efficacy of the cocktail against *Salmonella*.

Overall, our results suggest that the phages used in the study persisted differently in vivo and propagated differently on the challenge strain in vivo. This led to varying levels of the phages during infection and induced resistance to the most present phage. The limited in vivo replication of the majority of bacteriophages led to a significant initial decrease in *Salmonella* colonization but the overall effect was a delay in bacterial gut colonization.

Further analysis is needed to maintain a high level of all phages to optimize their efficacy on incoming *Salmonella* infection. To overcome these limiting parameters, phages may be encapsulated to be protected from gastric conditions as enhancing phage stability can result in higher quantities of phages administered on infection sites. Phages may be used before infection but also during the onset of infection to prevent the pathogen establishment and not only reduce but also eliminate it. The meta‐analysis conducted by Mosimann et al. (2021) suggested that the effects of phage treatment may be greatest within 14 days of treatment and in chicks before 14 days of age. Some authors also suggested that an early phage intervention may lead to better *Salmonella* reduction (Bardina et al., 2012; Borie et al., 2008). Indeed, covering the pre‐ and start of the infection may bring sufficient diverse phage before and during this key period for a successful viral infection. This early and brief administration of phages may eradicate the pathogen while limiting the development of resistance.

Phages are generally recognized as safe agents and, due to their high specificity toward the targeted bacteria are thought to induce dysbacteriosis of the intestinal microbiota. However, phages can have an indirect effect by lysing susceptible target bacteria, which could lead to cascading effects on other bacterial species, and thus modulate the gut microbiota (Hsu et al., 2019). Moreover, phage introduction during microbiome development may also induce some changes and regulate the gut microbiota of chickens (Febvre et al., 2019; Huang et al., 2022; Zhao et al., 2022). In that respect, investigating whether and how phage treatment could impact bacterial communities in vivo is a determinant, although not often reported in phage therapy studies. In this work, we showed that α‐diversity was not affected by phage treatment. The increase in α‐diversity over time observed in all groups reflects the gut microbiota development. Thus, no dysbacteriosis, in terms of loss of richness and equitability, was induced by the treatments. Clavijo et al. (2022) which is to date the only report on the effect of phage treatment in the gastrointestinal tract microbiota of poultry at a production scale, also demonstrated that phages do not affect the normal maturation of the microbiota.

Three of the four major phyla colonizing the intestinal tract of chickens were observed in our study, dominated by the Firmicutes as commonly found, whereas Bacteroidetes were not observed. In accordance with previous studies (Ranjitkar et al., 2016; Richards, Fothergill et al., 2019; Rychlik, 2020; Wei et al., 2013), the gut microbiota was dominated by *Lachnospiraceae*, *Lactobacillaceae*, *Ruminococcaceae*, *Enterobacteriaceae* families. However, PCA and β‐diversity analyses indicated that the microbiota composition of each group clustered separately throughout the trial, even at 6 days of age. This observation suggests that the highly variable microbiota development was dependent on either the treatment or the room environment. On one hand, microbial environment exposure during the first days of life could be one parameter that influences chicken's microbiota composition. Rychlik (2020) and Ludvigsen et al. (2016), for example, reported that chicks raised under the same conditions, but in separate rooms could cluster separately with different microbiota compositions. On the other hand, phages could also modulate microbiota composition and induce some variations. Considering the analysis of differentially abundant genera, a consistent observation is the enrichment of *Lactobacillus* and *Fusicatenibacter* genera and depletion in the *Flavonifractor* genus common in both phage‐treated groups. These mutual enrichments and depletion may be associated with phages. *Fusicatenibacter* are bacteria producing short‐chain‐fatty acids (SCFA) and *Lactobacillus* are lactic acid bacteria positively correlated to gut health. The increase of these genera may be the indirect result of phage lysis of *Salmonella* but also closely related bacteria such as *E*. *coli* allowing other bacteria to proliferate.

Overall, the changes that could be induced here by phages did not show deleterious effects on the gut microbiota, that is, in terms of microbial diversity, they rather seem to induce the enrichment of beneficial bacteria. Further analysis is required to confirm these microbial modulations by controlling influencing factors of the microbiome such as the environment. In any case, this work shows that analysis of the impact of phage treatment should be considered in the phage therapy strategy. Phage prophylaxis could be considered a promising strategy to control *Salmonella* infection in newly hatched chicks, especially when the infection load is low. Under these conditions, the strategy may eradicate the pathogen while avoiding the formation of resistance. A higher *Salmonella* infectious load is more challenging. In this case, a treatment at the end of production might be another strategic approach.

## AUTHOR CONTRIBUTIONS

**Lorna Agapé**: Conceptualization; methodology; data curation; investigation; validation; formal analysis; writing—original draft. **Pierrette Menanteau**: Conceptualization; methodology; investigation; writing—review and editing. **Florent Kempf**: Conceptualization; validation; formal analysis; data curation; visualization; writing—review and editing. **Catherine Schouler**: Conceptualization; validation; supervision; writing—review and editing. **Olivier Boulesteix**: Methodology; writing—review and editing; validation; resources. **Mickaël Riou**: Methodology; writing—review and editing; resources; validation. **Thierry Chaumeil**: Methodology; validation; writing—review and editing; resources. **Philippe Velge**: Conceptualization; validation; supervision; project administration; funding acquisition; writing—review and editing.

## CONFLICT OF INTEREST STATEMENT

Phages and part of the funding for this research originated from Lesaffre. The company had no involvement in conducting the experiments, analyzing the results, or writing the article.

## ETHICS STATEMENT

In vivo experimental infections were performed at the PFIE (UE‐1277 PFIE, INRAE Centre de Recherche Val de Loire, France, 10.15454/1.5535888072272498e12). The experiments with chickens were conducted in strict accordance with French legislation. All animal care and use adhered to French animal welfare laws. The protocols for this study were approved by the Loire Valley ethical review board (CEEA VdL, committee number 19) and the French Ministry of Education, Higher Education and Research (Ministère de l’Éducation Nationale, de l'Enseignement Supérieur et de la Recherche) under protocol No. APAFIS#24688‐202009081211981 v1. The principles of reduction, replacement, and refinement were implemented in all experiments.

## Data Availability

The raw 16S rRNA gene sequencing data generated and analyzed during the current study are available in the Sequence Read Archive (SRA) of the European Nucleotide Archive (ENA) under the accession numbers ERR10905515 to ERR10905713 of the BioProject PRJEB60023: https://www.ebi.ac.uk/ena/browser/view/PRJEB60023

## 1

**Table A1:** Lytic ranges of the phages for *Salmonella* serotypes.

Serotype	Total isolates	SalE_1	SalE_2	SalE_3	SalE_4	SalE_5	SalE_6	Phage Cocktail
4,[5],12:i:‐	7	0 (0%)	5 (71%)	7 (100%)	7 (100%)	7 (100%)	4 (57%)	7 (100%)
Agona	28	5 (18%)	15 (54%)	26 (93%)	28 (100%)	27 (96%)	9 (32%)	28 (100%)
Alachua	6	1 (17%)	0 (0%)	5 (83%)	5 (83%)	1 (17%)	1 (17%)	6 (100%)
Anatum	7	0 (0%)	0 (0%)	6 (86%)	7 (100%)	3 (43%)	1 (14%)	7 (100%)
Enteritidis	209	7 (3%)	201 (96%)	208 (100%)	208 (100%)	208 (100%)	195 (93%)	209 (100%)
Georgia	17	0 (0%)	17 (100%)	17 (100%)	17 (100%)	17 (100%)	17 (100%)	17 (100%)
Grampian	7	5 (71%)	0 (0%)	2 (29%)	6 (86%)	7 (100%)	1 (14%)	7 (100%)
Hadar	171	72 (42%)	13 (8%)	55 (32%)	171 (100%)	167 (98%)	80 (47%)	171 (100%)
Heidelberg	58	2 (3%)	53 (91%)	55 (95%)	57 (98%)	58 (100%)	48 (83%)	58 (100%)
Infantis	58	7 (12%)	1 (2%)	21 (36%)	57 (98%)	57 (98%)	18 (31%)	58 (100%)
Javiana	6	1 (17%)	5 (83%)	6 (100%)	6 (100%)	5 (83%)	5 (83%)	6 (100%)
Kentucky	33	31 (94%)	2 (6%)	33 (100%)	9 (27%)	21 (64%)	14 (42%)	33 (100%)
Livingstone	5	0 (0%)	0 (0%)	5 (100%)	5 (100%)	4 (80%)	2 (40%)	5 (100%)
Mbandaka	81	2 (2%)	2 (2%)	59 (73%)	58 (72%)	19 (23%)	8 (10%)	77 (95%)
Montevideo	19	0 (0%)	0 (0%)	18 (95%)	19 (100%)	18 (95%)	16 (84%)	19 (100%)
Muenchen	6	6 (100%)	0 (0%)	6 (100%)	6 (100%)	6 (100%)	5 (83%)	6 (100%)
Newport	38	24 (63%)	0 (0%)	36 (95%)	36 (95%)	33 (87%)	24 (63%)	38 (100%)
Ohio	20	1 (5%)	0 (0%)	20 (100%)	20 (100%)	20 (100%)	20 (100%)	20 (100%)
Oranienburg	6	0 (0%)	1 (17%)	6 (100%)	6 (100%)	5 (83%)	6 (100%)	6 (100%)
Reading	8	0 (0%)	8 (100%)	8 (100%)	8 (100%)	8 (100%)	2 (25%)	8 (100%)
Schwarzengrund	30	1 (3%)	19 (63%)	28 (93%)	26 (87%)	26 (87%)	24 (80%)	29 (97%)
Senftenberg	20	1 (5%)	1 (5%)	19 (95%)	19 (95%)	14 (70%)	7 (35%)	20 (100%)
Stanley	10	1 (10%)	5 (50%)	10 (100%)	8 (80%)	9 (90%)	7 (70%)	10 (100%)
Tennessee	8	0 (0%)	0 (0%)	8 (100%)	7 (88%)	7 (88%)	7 (88%)	8 (100%)
Thompson	103	71 (69%)	10 (10%)	103 (100%)	53 (51%)	95 (92%)	86 (83%)	103 (100%)
Typhimurium	262	8 (3%)	252 (96%)	249 (95%)	257 (98%)	245 (94%)	213 (81%)	260 (99%)
Westhampton	15	0 (0%)	0 (0%)	14 (93%)	15 (100%)	12 (80%)	10 (67%)	15 (100%)
